# Analecta

**Published:** 1842-03-05

**Authors:** 


### ANALECTA.

*Observations on the Incipient Stage of Cancerous Affections of the Womb.* By W. F. Montgomery, Professor of Midwifery to the King and Queen's College of Physicians in Ireland.—[Dr. Montgomery believes that the disease may be recognised at a much earlier stage than is usually thought, and that "it may, in many instances, be altogether turned aside, and the victim be rescued from the sad fate impending over her." There is no higher authority than our author in this matter, and the profession will be glad to receive such consolatory suggestions from him. The following is his description of the early period of the disease.]

*Symptoms.*—Sharp, but comparatively fugitive, lancinating pains in the back and loins, across the supra-pubic region, or shooting along the front of the thigh, or sometimes, along the course of the sciatic nerve, producing numbness, and not unfrequently debility of the whole limb.

In a large proportion of the cases, there is found a decided fulness, or a distinct tumour in one or other iliac hollow, with fixed pain, and tenderness traceable to, and, as it were, issuing out of the abdominal ring; there is, generally, more or less irritation of the bladder, with dysuria, and the patient often complains of a sensation about the lower part of the rectum, which induces her to think that she is labouring under piles. Menstruation, though in some instances disturbed, is, much more frequently, quite regular in its returns, but there is apt to be bursts of hæmorrhage, either accompanying the discharge, or occurring in the intervals: there is little or no leucorrhœal or serous discharge, often none ; and it is not until the disease has existed for a considerable time, that the appetite is impaired, sleep is disturbed, the flesh becomes softer and wastes, and the countenance pale, and expressive of distress.

*Examination per Vaginam.*—The margin of the os uteri is found hard, and often slightly fissured, and projects more than usual, or is natural, into the vagina, and is irregular in its form.

In the situation of the muciparous glands, there are felt several small, hard, and distinctly defined projections, almost like grains of shot, or gravel, under the mucous membrane. Pressure on these, with the point of the finger, gives pain, and the patient often complains that it makes her stomach feel sick.

The cervix is, in most instances, slightly enlarged and harder than it ought to be. The circumference of the os uteri, especially between the projecting glandulæ, feels turgid, and to the eye, presents a deep crimson colour, while the projecting points have sometimes a blueish hue.

In two cases of women who died, one of fever, and the other of pneumonia, in a more advanced stage of this condition of the os uteri, the substance of the uterus was found considerably increased in size and thickness, and was intensely vascular.

There is no thickening, or other alteration of structure in any part of the vagina, at its conjunction with which, the cervix uteri moves freely ; nor is there any consolidation of the uterus with the neighbouring contents of the pelvis ; in fact, the morbid organic change appears to be, at first, entirely confined to the os uteri, and lower portion of the cervix.

This stage of the affection is, in many instances, *very slow*, lasting, sometimes, *for years*, before the second and hopeless stage is established ; during this time, the patient experiences only comparatively slight and transient attacks of pain, or perhaps only sensations of uneasiness, referred often to the situation of one or other of the ovaries, or about the os uteri, with anomalous tingling along the front and inside of the thighs ; these last for a few hours, or a day or two, and then disappear, perhaps for weeks, but again and again return in the same situation, and, for a long time, are not increased in severity ; the patient finds that sexual intercourse now occasionally causes her pain, which she ascribes to some deep-seated part being touched, and the act is followed by an appearance of blood ; she is, also, often troubled with slight irritability of the bladder ; but the appetite, digestion, and sleep may, for a long time, continue good, and the pulse generally gives no indication of the existing disease or its changes ; an observation which will be found applicable to many uterine affections of a very grave character ; in short, the general health may long remain quite undisturbed, nor has the patient, in many instances, the slightest suspicion that there is any thing seriously wrong with her, nor thinks of seeking for medical aid until she is induced to do so by the solicitations of her husband, or some anxious friend who has become, as she thinks, unreasonably alarmed about her state.

In not a few instances, I have known the first indication of ill health to have been pain, affecting the muscles of the back or extremities, and so closely resembling rheumatism as to pass for that disease. In a case of this kind which I saw in Thomas-street, in consultation with Mr. Smyly, no suspicion of uterine disease had been entertained, previous to his seeing the patient, until alarm was excited by a profuse hæmorrhage, and, on examination, carcinoma was found fully established.

In another case, which I was brought down to see in the County Mayo the first uneasiness so closely resembled sciatica, that the lady had been, for two years undergoing treatment for that affection, before the existence, of cancer uteri was suspected, and then, the disease was found far advanced.

It very often happens, that the pain connected with carcinomatous affections of the uterus recurs periodically, and exactly at the same hour of the day, and thus, so far, assumes the character of mere nervous or neuralgic complaints, independent of organic disease, and, in consequence, valuable time has been lost, and the appropriate treatment omitted; and all this, because the proper investigation was not instituted at first.

Sometimes, both patient and practitioner are deceived, as to the real source of the symptoms, because these happen to be only perceptible in the deranged functions of other, and perhaps, remote organs; for instance, nothing is more common than for patients to apply for advice on account of irritability of the bladder, or as they often call it "the gravel," where the disturbance of that organ is, on examination, found to be only sympathetic with morbid alteration in the functions, or organization of the uterus; thus, also, I have known œdema of one limb, or swelling of the inguinal glands, the first complaint, for which the patient sought advice, quite unconscious and unsuspicious of any uterine disease; in one instance, after the continuance for some months, of the condition just alluded to, the uterus was found extensively and hopelessly diseased, and even quite immovable from consolidation with the surrounding parts.

When patients thus affected do take the alarm, and apply for advice, it is much to be lamented, that their statement is too often received as sufficient grounds for a merely palliative line of treatment, and *their symptoms are prescribed for*, without any examination being instituted, to determine the exact state of the uterus, and ascertain whether there have taken place any organic alteration or not. I am satisfied, that if the very contrary mode of proceeding were adopted, and a careful vaginal examination made the preliminary step in all such instances, and a decided plan of treatment at once adopted and persevered in, many a victim would be snatched from the horrors of a life where agony is measured by years, and death comes invested with the direst tortures that our "flesh is heir to."

*Pathology.*—Sufficient observation has fully satisfied me, that, in the great majority of instances, the first discoverable morbid change which is the forerunner of cancerous affections of the uterus, takes place in, and around the muciparous glandulæ, or vesicles, sometimes called ova nabothi, which exist in such numbers, in the cervix and margin of the os uteri; (see my work on Signs of Pregnancy, &c., pl. ix. fig. 2;) these become indurated by the deposition of scirrhous matter around them, and by the thickening of their coats; in consequence of which, they feel *at first* almost like grains of shot or gravel under the mucous membrane, afterwards, when they have acquired greater volume by further increase of the morbid action, they give to the part the unequal, bumpy, or knobbed condition like the ends of one's fingers drawn close together. When this *second stage* (usually described by writers as the first) is established, all means hitherto devised have failed in producing any permanent beneficial effect.

It might, at first sight, appear an objection to the above view, that cancer uteri sometimes commences in the upper parts of the organ, or even in its appendages, where these muciparous follicles, or ova nabothi are not generally supposed to exist; but that they do exist in these situations, and occasionally appear there very distinctly, I have repeatedly ascertained, and demonstrated, and have preserved several specimens of them fully developed in these parts; this observation has been made by many others long since (see Morgagni, Epist. 47, No. 20, *et seq.*;) their detection in a state of developement, in the latter situations, is however, a comparatively rare occurrence.

With regard to the pain and tenderness, with fulness, and sometimes a distinct tumour, more than once already alluded to as felt in the iliac hollow, I wish to observe, that this affection of either the ovaries or the glands at the sides of the uterus, in different forms and stages of carcinomatous affections of the organ, is a much more constant occurrence than I think is generally supposed; and moreover, I am, from repeated observation, much inclined to believe that it is often the source from which the morbid irritation originally springs, and is communicated to the uterus; it will be seen that it was observed in three out of four of the cases described in this paper; it existed in four out of five cases recently seen by me in advanced states of the disease, and of twelve specimens preserved in my museum, it is observable in every one. I may add, that I feel no doubt that early attention to this symptom, and the adoption of *decided measures*, suited to its removal, would in many instances, in which as yet no distinct indication of uterine disease can be detected, save the patient from the future occurrence of such a dreaded calamity; I believe this to be one of those contingencies, in which if we do not extinguish the spark, we may be afterwards unable with all our efforts, to quench the flame.

*Diagnosis.*—The only affection of the uterus, for which this disease could be mistaken, and that only by carelessness, is the irritable uterus; from which, however, it is essentially different; inasmuch as it is accompanied by, and tends to produce still further change in the structure of the organ; which, although unduly sensitive under examination, is not the seat of the *exquisite* tenderness and pain observed in irritable uterus; from which it also differs in having the increase of volume of the parts affected well marked, and constant, until removed by treatment, and in the existence of the other organic alterations already enumerated, as well as in the different result of the affection. From the second, or fully formed stage of cancer uteri, any one accustomed to examine the organ must at once distinguish it.

*Treatment.*—In almost every instance, the treatment should be begun by *the local abstraction of blood*, either by cupping, or by leeches applied directly to the os uteri, or as near as possible to the organ; and their application will in most cases, require to be frequently repeated, and should be accompanied by the free use of anodyne fomentations. With regard to *venesection*, although it may be desirable to practise it under particular circumstances, it is not in general required; and I would say that the case in which it is called for, should be regarded as an exception in the plan of treatment generally most suitable.

Except there be something specially to forbid its use, *mercury* should be given in some form, so as to bring the system very gently, but decidedly, under its influence; for which purpose, it may be combined with iodine in very minute proportions, with camphor, opium, hyosciamus, or hemlock; and occasionally by friction, especially where there exists evidence of inflammatory action in the iliac hollow, as already adverted to.

Afterwards *iodine* or *hydriodate of potash* may be used both internally and externally; and *iron* will be found a most beneficial and powerful agent, especially in the form of the saccharine carbonate, or the carbonate given in the nascent state.

The *iodide of iron*, which combines to a certain degree, the powers of both remedies, may also be used with advantage in most cases, and will be best administered in the form of *Dupasquier's syrup*, which is now prepared, of different strengths, by our chemists and apothecaries.

*Arsenic* has received the testimony of many able practitioners in its favor, as an agent capable of giving great relief in these affections; and I can add mine to the same effect, having obtained marked benefit from its use, especially when combined with anodynes, even in advanced states of this disease: of *iodide of arsenic* I cannot speak from experience; but I think that both from the nature of the compound, and still more from the success which appears to have attended its administration in cancerous affections by Dr. A. T. Thompson and Dr. Crane, of Canterbury, we are justified in expecting that it will prove a useful remedy in such diseases.

*Counter-irritation* is an agent of great influence in this complaint, and may be established in a variety of ways, which it is unnecessary to enumerate; but a very effectual mode, is by making a small blister over different parts in succession, and keeping it discharging freely for several days, by the application of the French dressing, or Albespyer's papers.

The *warm bath* and the warm *hip bath* are means of great value throughout the treatment of this affection; and their effect in soothing the uterine irritation may be much promoted by admitting the warm water into contact with the internal surface of the vagina and os uteri, which may be accomplished without difficulty, by introducing into the vagina one of Lassaigne's speculums, which are made of wire-gause coated over with caoutchouc; or a small plain metal speculum, with perforations in its sides, will answer the purpose extremely well; and the patient can apply the instrument for herself, better, indeed, than any one else could. I may observe that where warm baths are used in the treatment of amenorrhœa, this mode of managing them may be adopted with great advantage.

After the removal of the congestion and organic changes from the os uteri, there remains, occasionally, a sensitiveness of the part, which causes the patient much discomfort, and which will be best relieved by the use of the bath, as above directed; conjoined with anodyne applications to the part, or nitrate of silver in solution; the best mode of applying which, is by means of a bent glass tube, of about an inch in diameter, which the patient can introduce and manage for herself; all that is necessary is, that she should lie on her back, and introduce the tube as far as its curvature, and then pour into the upper end the medicated solution, which will immediately pass to the os uteri, and can be retained there as long as is necessary, the tube filling the vagina sufficiently to prevent its flowing away, which is a great advantage, above all other methods with which I am acquainted for applying lotions to this part.

The patient should be strictly enjoined to avoid every thing that could stimulate the uterus, such as riding on horseback, &c.; but, especially, she should refrain from sexual intercourse. I need scarcely add, that the greatest care and moderation will be essentially requisite in the quality and quantity of the patient's diet. Wine, if used at all, should be of a very mild kind, and very sparingly taken; and the same rule should apply to malt drinks; the stronger kinds of ale and porter should be altogether prohibited.

No circumstance connected with the treatment of this affection requires more scrupulous attention than the regulation of the patient's habits and mode of living; indeed, if this be not very carefully managed, all other measures will most probably be defeated.

This is, perhaps, of all others, the case in which extirpation of the part might be expected to be successful; but I could not recommend it, because the operation is a very formidable one, and I know the affections to be curable without it; besides, we have no means of accurately determining whether the taint is really thus isolated, or whether other parts are not already contaminated; so that we run the chance of only obtaining that equivocal triumph in which an operation is blazoned forth as being crowned with brilliant success, while the patient dies of the disease for which it was performed.

[These remarks are followed by cases, which we have not space enough to extract from Dr. M.'s interesting paper. The following propositions are his final conclusions.]

1. That the affection here described, is the first stage of cancer uteri.

2. That its existence is indicated by symptoms and organic changes sufficiently marked to attract our attention, and cause its discovery on examination.

3. That, if not arrested *promptly* and *decidedly*, it will pass into an incurable condition.

4. That it has been, and therefore can be, so arrested and cured by suitable treatment, and the patient saved from the lingering agony to which she must otherwise fall a victim.

[The author concludes with reminding the physician that he should not disclose to the patient, unnecessarily, his view of the nature of the disease. That is, the patient may know that the disease is formidable, or even incurable, but she will shrink from the term cancer.]

*Dublin Journal.*

---

*Peculiar Nervous Affection following Parturition.*—In the Westminster Medical Society, session of Nov. 13th, 1841, Mr. Lavies related some particulars of two cases of peculiar nervous affection succeeding parturition. In one the lady had been delivered of her second child, two or three weeks afterwards, she was affected with a tetanic condition of the muscles of the face on the left side; this was removed by purgation, but the muscles of the opposite side of the face became affected in the same way. Purgatives also succeeded in this case in removing the affection; several members of the body suffered in a similar manner, without any symptoms of fever being present. The phenomena gave way under the use of full doses of laudanum and antimonial wine. In the second case the woman was in labour with her second child. Immediately the index finger touched the os uteri, a peculiar kind of pain, but not like cramp, was experienced down the inside of the leg. This pain in the leg lasted for some weeks after delivery, but came on only when she moved. When quiescent there was no pain, but the movement of the great toe produced it. She suffered for many weeks with it; remedies were of no avail. It wore itself out at last. She had not suffered in the same way with any of her subsequent children.

*London Lancet, Nov.* 20, 1841.

*Case of acute Laryngitis, with remarks on Bronchotomy.* By Jordan Roche Lynch, M. D.—John Whaler, aged 58, a laborer, lodging at 56, West-street, a very powerful man, of temperate habits, and latterly had suffered serious privations from want of employment. He was attacked after exposure to wet, about noon on the 3d instant, with pain, tenderness, and a sense of constriction of the larynx. He went home, and applied in the evening for a relieving-officer's order for medical assistance. I attended on its receipt, and found him laboring under all the symptoms of acute laryngitis. The face flushed; the skin hot; the pulse full, and hard; countenance anxious in the extreme; the eyes staring; the nostrils raised; the voice reduced to a whisper, and articulation very difficult; the long deep shrill, stridulous inspiration so marked as to be heard outside the room, so characteristic, that if once heard it can never be forgotten. On examination of the fauces, there was no appearance to indicate the extent of mischief going on; great tenderness of the larynx. He was bled to sixteen ounces. The blood dark, thick, and flowed *pleno rivo.* As he had been reduced by insufficient nutriment, I was cautious as to the quantity abstracted. He was ordered calomel, ʒss; opium and tartarised antimony, of each, gr. xj. Divide into six powders, and give one every half hour. I saw him again in an hour: no relief; the act of swallowing now caused convulsive fits of coughing, with frequent attempts to hawk up a thick ropy mucus; difficulty of breathing increased; very restless, and every three or four minutes changing his position, in the vain hope of procuring ease; if he tried to lie down, he started up immediately gasping for breath, and in the greatest agitation; respiration every moment becoming a more convulsive struggle. He was again bled to fifteen or sixteen ounces; at which he expressed some mitigation. The dysphagia was, however, so great, that he could not swallow a drop of water. * * *

[Dr. L. then ordered twelve leeches and mercurial unction to the axillæ, and called to the case Mr. Waltham, house-surgeon at St. Bartholomew's, who found him with a pulse soft and full, profuse diaphoresis, and the breathing and other symptoms as urgent as before. He was then removed to the hospital.]

On admission he was seen by the resident medical officer, Mr. Hurlock, who prescribed the warm bath, with twenty leeches to the throat; calomel, ℈j; tartarised antimony, gr. iij. To be divided into six doses, one to be taken every half hour.

At ten o'clock the next morning there was not the slightest amendment; on the contrary, he was much more depressed; the difficulty of breathing continued in the same state as when he was admitted. At eleven o'clock Dr. Roupell saw him, and promptly decided upon the necessity of an artificial opening to his lungs being made; but before the arrival of the surgeon, which was not more than an hour and a half (!) after the decision, a great change for the worse came on: so that while his trachea was being opened he was expiring. Artificial respiration was kept up for some minutes after the operation.

I attended the autopsy: Dr. Roupell gave a short and very accurate history of the case. Mr. Paget carefully removed the pharynx and larynx. The following appearances presented:—The epiglottis erect, thickened, in such a state as to be incapable of protecting the glottis from the contact of matter passing into the pharynx; the mucous membrane of an uniform dark-red color; the folds forming the rima glottidis very much thickened, and effusion into the sub-mucous tissue, and beneath the fine lining membrane which is reflected over the thyro-arytenoid ligaments, and which sinks in between the superior and inferior ligaments, forming the ventricle of the larynx: in this situation the matter effused appeared to be sero-purulent. On bringing the sides of the chink of the glottis in contact, the natural opening was completely obliterated by the thickening, which prevented respiration and was the obvious cause of death. There was no false membrane; this inflammation did not extend down the trachea; the lungs were in the lower portion of the lobes filled with dark blood, and in the superior filled with air, but not emphysematous: it was clearly that form of disease to which the term laryngitis is properly restricted.

*Remarks.*—The prognosis is always unfavourable: Dr. Cheyne considered it the most fatal of all inflammations. The only hope is by giving relief by the operation. Upon this point there is great difference among medical authorities. Dr. Baillie was of opinion that blood-letting is of no use, and that tracheotomy should not be resorted to in less that thirty hours. In this case death put an end to the patient's sufferings in less than that time. Dr. Armstrong mentions two cases that terminated fatally in seven and eight hours. Mr. Lawrence says, "that bronchotomy should be done as soon as the symptoms enable us to decide upon the nature of the disease;" and wisely makes the condition of the sufferer more the criterion than the period of the attack. Louis observes, that as long as bronchotomy is considered an extreme measure, it will be performed too late. Drs. Macintosh and Elliotson are in favour of the operation, but recommend previous trial of venesection and mercury. This is very good advice in the first stage, before effusion has taken place.

In such a fell and insidious disease, when the sentinel of the portal of life is being overpowered, delay is death; and most assuredly the danger from the operation cannot be compared with the danger from the obstruction to the breathing which it is calculated to remove: it is of no use whatsoever to have recourse to it when the face becomes livid, and the faculties obtuse, from the circulation of black blood in the brain.

The operation of bronchotomy is less likely to add to the irritation, than that recommended between the thyroid and cricoid cartilages; it is also of a more simple nature: it is likewise further removed from the seat of inflammation, and the mucous membrane is here endued with less sensibility.

Some time ago, in a case in some respects similar to this just detailed, I cut down in the early stage upon the trachea in the manner described by Mr. Lawrence in his lectures, and introduced a very short canula nearly approximating to the shape of that of M. Bretonneau (a description of which is given in the 25th plate, fig. 4, of M. Bourgery;) and persevered with calomel and opium, and the application of leeches to the fauces; and although the canula caused great irritation, the opening closed about the eighteenth day, and the man recovered.

In every case, as soon as the disease has fully manifested itself, bronchotomy should be performed, or an excision of one or two of the rings of the trachea, as described by Crampton. The tenderness of the larynx, and the peculiar inspiration quite distinct from the hoarseness that often accompanies severe bronchitis, and which is caused by mucous or catarrhal inflammation, are the two pathognomic signs which justify the use of the knife, and afford to your patient the only certain means of saving his life. Medical men are, by their exposure to vicissitude of temperature, peculiarly liable to this complaint, several of the greatest eminence have died of it; they should be always mindful of the saying of an old and great authority—"Celerrime intereunt, cynanche laryngea laborantes nonnunquam et antiquam medicum accersiverent.—*London Lancet*, *Nov.* 27, 1841.

---

*Spontaneous Dislocation of the hip-joint after labour.* By J. Nottingham, late House Surgeon to the Liverpool Infirmary.—August 27, 1841, I was requested to visit a poor woman, the wife of a labourer in Liverpool, 36 years of age, formerly healthy and strong, and, from her account, belonging to a family presenting none of the characteristics of scrofula, or any hereditary form of disease. Having spoken of other complaints, she said that her hip had been out since her last confinement.

On examination I found the head of the right femur on the dorsum of the ilium, with considerable shortening of the limb; the heel of the affected side being about three inches from the ground when she stands on the opposite foot.

She was married at the age of 18, and had her first child between her 19th and 20th year; has borne nine children, and had one miscarriage of four months.

Her last child, a boy, lived eleven weeks; was born sixteen months ago, after a labour which continued thirty-six hours.

On the 4th day after confinement she had great pain in the abdomen, and difficulty in voiding the urine. Leeches were applied, and such other measures adopted as circumstances seemed to demand.

On the ninth day after her confinement she sat up during an hour and a half; and on the tenth day was attacked by great pain in the right hip, which continued for several days, and for which leeches were applied.

From her account it would seem that some time elapsed before the head of the femur got into the position which it now maintains.

The patient is quite sure that she never had the slightest uneasiness in the hip before her confinement, and had nothing to complain of during her pregnancy saving a varicose state of the veins of the lower extremities.

From the time and manner in which the accident occurred, we can scarcely fail to think that some effect produced by parturient efforts on the bones of the pelvis, was the forerunner of the malady. That the case is unusual, and not easily elucidated, may be regarded as the apology for this communication. The two sides of the pelvis are on the same level; and there is nothing to lead us to suppose that disruption of the pubic or of the sacro-iliac symphysis had ever taken place.

The patient is again six months advanced in pregnancy.—*London Med. Gaz.*, *Nov.* 19, 1841.

---

*The Medical Examiner* is published every Saturday by *J. C. Turnpenny*, N. E. corner of Spruce and Tenth streets. Terms—*three dollars* a year for one copy, or *five dollars* for two copies sent to the same post office, in all cases in advance. Postage the same as on newspapers. Letters to be addressed,—post-paid,—to the Editors of the Medical Examiner, Philadelphia.

*Reynell Coates, M. D.*, Acting Editor.

*J. B. Biddle, M. D.*, and *W. W. Gerhard, M. D.*, Co-editors.

*J. B. Biddle, M. D.*, Proprietor.

